# Exploring the standardization of human nasal antibody measurements

**DOI:** 10.1080/22221751.2025.2475822

**Published:** 2025-03-12

**Authors:** Xuanxuan Zhang, Yulong Fu, Si Chen, Guanxing Liu, Ying Wang, Qian He, Qian Wang, Na Li, Zhongfang Wang, Ling Chen, Junzhi Wang, Zhenglun Liang, Miao Xu, Qunying Mao

**Affiliations:** a State Key Laboratory of Drug Regulatory Science, Evaluation of Biological Products, Key Laboratory of Research on Quality and Standardization of Biotech Products, Institute of Biological Products, National Institutes for Food and Drug Control, Beijing, People’s Republic of China; b Research Units of Innovative Vaccine Quality Evaluation and Standardization, Chinese Academy of Medical Sciences, Beijing, People’s Republic of China; c School of Life Science and Biopharmaceutics, Shenyang Pharmaceutical University, Shenyang, People’s Republic of China; d Drug and Vaccine Research Center, Guangzhou National Laboratory, Guangzhou, People’s Republic of China; e Guangzhou Institute of Infectious Disease, Guangzhou Eighth People’s Hospital, Guangzhou Medical University, Guangzhou, People’s Republic of China; f Changchun Institute of Biological Products Co., Ltd., Changchun, People’s Republic of China; g Beijing Minhai Biotechnology Co., Ltd., Beijing, People’s Republic of China

**Keywords:** SARS-CoV-2, nasal antibody, binding activity, national standard, commutability

## Abstract

Mucosal immunity is crucial for preventing the infection and transmission of respiratory viruses. Nasal antibody is inversely correlated with a lower risk of infection with respiratory viruses. However, the current reference standard for nasal antibody assessment is serum-based, mainly consisting of monomeric IgG and IgA. The applicability of serum-derived standards for assessing nasal antibodies, consisting mostly of dimeric or polymeric secretory IgA (sIgA), remains unvalidated. Herein, we first proved that the sera-derived standard was not applicable for assessing nasal antibodies. Using a non-homologous standard as a calibrator introduced systematic error up to 10 times, which did not benefit the understanding of mucosal antibody response. Therefore, we attempted to develop two candidate standards (CS1, CS2) using nasal mucosal lining fluids (NMLFs) collected from SARS-CoV-2 Omicron convalescents or intranasal vaccine recipients, and CS3 using a sIgA monoclonal antibody. CS2 exhibited broad-spectrum binding activity against 12 SARS-CoV-2 strains, including all tested Omicron subvariants. A collaborative study conducted by seven laboratories demonstrated that CS2 improved the harmonization of inter-laboratory variability (pre-standardization geometric coefficients of variance, 14–314%; post-standardization, 3–35%). Using CS2 ensured an accurate assessment of nasal antibodies. Thus, CS2 was established as a national standard for evaluating nasal SARS-CoV-2-specific antibodies (Lot: 300052-202401, 1000 U/mL). Our work provides a benchmark for evaluating mucosal vaccines for SARS-CoV-2 and inspires new avenues for developing new reference standards for other mucosal vaccines.

## Introduction

Respiratory viruses, such as SARS-CoV-2, primarily replicate in the respiratory epithelial cells and pose a substantial threat to global public health. SARS-CoV-2 has infected 776 million people and caused 7.05 million deaths worldwide [1]. The virus initially infects epithelial cells in the nasopharynx using the receptor-binding domain (RBD) on the spike protein to interact with the angiotensin-converting enzyme 2 (ACE2) receptor. The spike protein, especially the RBD segment, has been recognized as the target for antibody and vaccine countermeasures. Vaccination is an effective and cost-efficient approach to controlling infectious diseases. Since the approval of the first SARS-CoV-2 vaccine for emergency use at the end of 2020, 13.64 billion doses have been administered globally [2]. Mass intramuscular vaccination reportedly reduced COVID-19 severity and mortality; however, these vaccines were ineffective in preventing infection and blocking the transmission, especially the Omicron subvariants that primarily infect and replicate in the upper respiratory tract. The efficacy of a fourth dose of mRNA vaccine in preventing symptomatic Omicron infection was only 11–30% one month after vaccination [3]. Intramuscularly administered SARS-CoV-2 vaccines can induce systemic immune responses but not respiratory mucosal immune responses [4, 5]. The antibody response, measured by spike or RBD binding IgG titres and neutralizing antibody titres against pseudoviruses or authentic SARS-CoV-2 variants, has been used as the most important parameter for assessing immunogenicity and vaccine-induced immune response. Therefore, in the early stage of SARS-CoV-2 pandemic, WHO established the first international standard (IS, 20/136) using a pool of convalescent plasma from 11 patients infected with the ancestral strain (wild-type, WT) in 2020 [6]. This standard has been distributed to many companies and organizations that participated in developing SARS-CoV-2 vaccine. The blood-neutralizing titres reportedly correlate with vaccine efficacy in preventing symptomatic infection caused by WT [7, 8]. However, with the emergence of Omicron subvariants, blood-neutralizing titres showed poor correlation with symptomatic infection caused by Omicron subvariants [9, 10]. Increasing studies have demonstrated that mucosal antibodies and cell-mediated immune response in the upper respiratory tract are critical in preventing SARS-CoV-2 infection and transmission [11–13]. Higher levels of spike-specific secretory IgA (sIgA) in the nasal mucosa are associated with lower Omicron breakthrough infection [14]. Hence, understanding the characteristics and extent of mucosal immunity, especially mucosal antibody response, can facilitate the evaluation and development of mucosal vaccines.

Unlike IgG which accounts for about 80% of antibody isotypes in the blood and with 15% monomeric IgA and 5% IgM, sIgA exists mostly as dimeric and multimeric forms with a secretory component and constitutes about 86% of antibody isotypes in the mucosa of the upper respiratory tract, while IgG accounts for only about 14% [15]. Purified nasal sIgA showed 1–2 orders of magnitude greater potency than serum IgG and IgA in binding to spike proteins and neutralizing SARS-CoV-2 variants [15]. Intranasal instillation of purified nasal sIgA protects mice against the challenge of Omicron subvariants, whereas instillation of the same amount of serum IgG or IgA failed to exert the same protection [16]. Therefore, specific sIgA may be used as a key indicator for evaluating the effectiveness of mucosal vaccines. However, there is only one reference standard provided by WHO which was derived from blood samples collected from convalescents during the early pandemic. Because of the difference in antibody isotypes and monomeric versus polymeric compositions between blood and mucosal fluids, a blood-derived sample may not be suitable as a reference standard for assessing mucosal antibodies. In this study, we first characterized the composition of immunoglobulins (Igs) in nasal mucosal lining fluids (NMLFs) and serum samples. In addition, the applicability of several standard serum-, NMLFs-based standards in nasal antibody assessments was evaluated. A standard for assessing nasal antibodies to SARS-CoV-2 was established, which will be useful for developing nasal vaccines for SARS-CoV-2.

## Materials and methods

### Ethics statement and human subjects

This study was conducted with the approval of Guangzhou Eighth People’s Hospital (No.202303240). NMLFs were obtained from SARS-CoV-2 convalescents or intranasal vaccine recipients. All donors provided informed consent for the use of their samples. The clinical information of donors is in Table S1.

### Western blot (WB) analysis

Purified nasal sIgA, human serum, and purified serum IgG were used as WB standards. Horseradish peroxidase (HRP)-conjugated goat anti-human IgA heavy chain (HC) (Abcam, UK), goat anti-human IgG Fc (Abcam, UK), mouse anti-human IgM (SouthernBiotech, USA), goat anti-human IgE (Invitrogen, USA), and goat anti-human IgD heavy chain antibodies (GeneTex, USA), were used to detect IgA, IgG, IgM, IgE, IgD. After adding chemiluminescent HRP substrate (Millipore, USA), polyvinylidene fluoride (PVDF) membranes were detected by Bio-Rad imaging system.

### Electrochemiluminescent (ECL) assay

To characterize the dose–response curve of NMLF or serum, samples were diluted in a 4-fold dilution series with proper initial dilution fold and detected by the V-PLEX SARS-CoV-2 Panel 33 (IgA) kit (Meso Scale Diagnostics, Maryland, USA), following the manufacturer’s instructions.

### Preparation of candidate standards (CSs) and collaborative samples

The WJ-XBQ06 nasal irrigator (Andon, Tianjin, China) containing saline was adjusted to the lowest speed. Donors slightly tilted their heads, blocked one nostril with the irrigator nozzle, and put a 50-mL centrifuge tube under the other nostril. With breath hold, the saline entered one nostril and exited the other. 250 ml of NMLFs were collected from each donor in the morning and evening for 2 days. NMLFs were centrifuged at 12,000rpm for 20 min to collect the supernatant. Then 100-kD ultrafiltration tubes (Merck Millipore) were used to concentrate NMLFs. Before pooling to development CSs, NMLFs were evaluated to ensure that they did not contain 14 common pathogens, namely SARS-CoV-2, respiratory syncytial virus, influenza A virus, influenza B virus, *Haemophilus influenzae*, human metapneumovirus, *Klebsiella pneumoniae*, *Streptococcus pneumoniae*, *Mycoplasma pneumoniae*, *Bordetella pertussis*, *Moraxella catarrhalis*, rhinovirus, herpes simplex virus, and enterovirus. CS1 and CS2 were mixtures of NMLFs collected from 7 and 11 donors, respectively. In addition, a monoclonal antibody with broad binding activity was used to prepare CS3 (Table S2). CS2 and CS3 were freeze-dried in a LYO-0.5 freeze drier. Primary drying was performed at −20°C for 40 h at 0.1 mbar followed by 0°C over 4 h. Secondary drying was performed at 10°C for 2 h at 0.2 mbar and 30°C for 6 h.

This collaborative calibration study included ten samples (Table S3).

### Participants and assay methods

Seven laboratories with experience in anti-SARS-CoV-2 mucosal sIgA detection participated in this study (Table S4). All laboratories were randomly allocated code numbers, 1 to 7.

Table S5 summarizes the assays used by the participants. The main assay was ELISA. Lab 7 performed ECL assay based on the manufacturer’s instructions. Participants were asked to perform three independent assays on different days.

Lab 7 tested a panel of 40 samples using ECL and ELISA (anti-WT-RBD). Thirty of them were native human nasal samples. Both the undiluted and 16-fold diluted CS1, CS2, and three serum samples were also assessed using ECL and ELISA.

### Statistical analysis

CS1 (coded 24001), CS2 (coded 24002), and CS3 (coded 24003) were used as reference standards for analysis. Potency estimates relative to the sample selected as the standard were calculated using a parallel line analysis or sigmoidal curve model with log-transformed responses [6]. For parallel line analysis, a minimum of three dilutions were ensured in the line section of the assay response range. Samples with a linear r^2^ value below 0.90 were excluded [17]. Test samples with response ranges that did not overlap with the range of reference standards or those non-parallel with reference standards were also excluded. Acceptable criteria for parallelism were defined as slope ratios of the coded duplicate samples relative to each other (24001 relative to 24004, 24002 relative to 24005, and 24003 relative to 24006) [17].

All valid data were used to generate geometric mean titres (GMTs) or potencies for each laboratory, and these laboratory means were used to calculate the overall geometric mean (GM) [18, 19]. Variability between assays and laboratories has been expressed using geometric coefficients of variation (GCV = (10^S^-1) × 100%, where S is the standard deviation of the log10 titre or potency estimates) [6, 17, 19]. The GCV was not calculated based on results supplied by a single laboratory. Microsoft Excel 2016 (Microsoft Corporation) was used for the calculations and analyses.

To assess the level of agreement between all pairs of laboratories, Lin’s concordance correlation coefficients were calculated using log titres or log potencies for the collaborating samples, excluding the serum sample (24009). Calculations were performed using the R package “DescTools.”

For commutability assessment, the ELISA and ECL measurement results of NMLFs and serum samples were logarithm-transformed. The transformed data of 30 native human nasal samples were analysed with Deming regression and 95% prediction intervals.

### Stability studies

CS1 was stored at −150°C, −80°C, 4°C, 25°C, and 37°C for up to 78 weeks. CS2 and CS3 were stored at −150°C, −20°C, 4°C, 25°C, 37°C, 45°C, and 56°C for up to 48 weeks. CS1 was frozen and thawed thrice to assess its in-use stability. In addition, the stabilities of CS2 and CS3 reconstituted in the liquid form were assessed. The reconstituted CSs were stored at 4°C for up to 14 days. The potency of all stability samples relative to the baseline, samples stored at −150°C, were assessed in triplicate using ELISA.

## Results

### Characterizing the composition of Igs in NMLFs and serum

**Figure 1.**
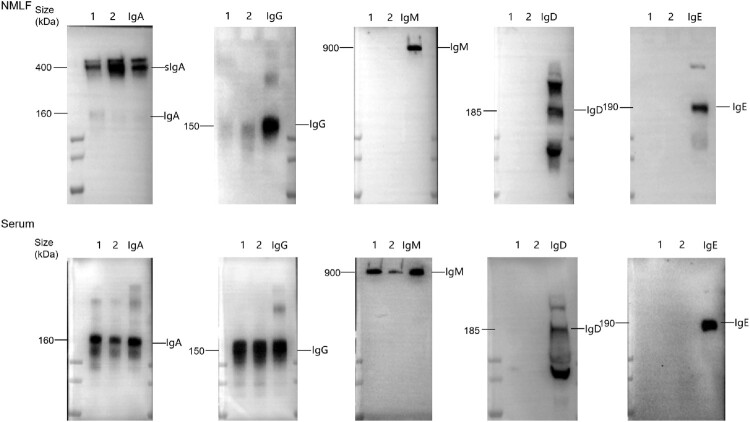
Characterizing the composition of immunoglobulins (Igs) in NMLFs and serum. Purified human sIgA, human serum, and purified serum IgG were used as references.

We first used WB to assess the Ig composition of NMLFs and serum. Unlike serum IgA, which exists in monomeric forms with molecular weights of approximately 160 kDa, nasal mucosal sIgA dominantly exists in dimeric and multimeric forms with molecular weights around 400 kDa or higher (Figure 1). Monomeric IgG with molecular weights of approximately 150 kDa was detected in NMLFs but only in a small amount. Additionally, no IgM, IgD, and IgE were detected in NMLFs (Figure 1). In contrast, serum Ig predominantly consisted of monomeric IgG, with a few IgM and monomeric IgA. IgD and IgE were not detected in the serum.

### Evaluating the applicability of serum-derived standards for assessing nasal SARS-CoV-2 RBD-specific antibodies

**Figure 2.**
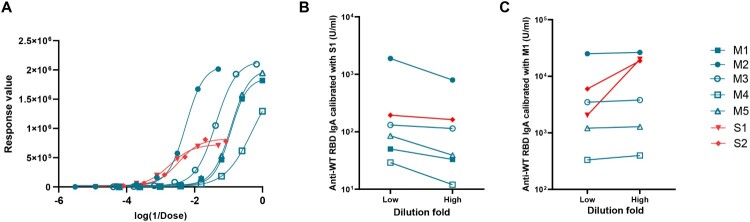
Applicability of serum standard in mucosal samples. (A) Dose-response curve for various samples using the ECL method. (B) The impact of sample dilution factor on quantification results when using S1 as the reference standard. (C) The impact of sample dilution factor on quantification results when using M1 as the reference standard. The anti-SARS-CoV-2 WT RBD IgA concentrations of S1 and M1 were defined as 1000 U/ml. Detection values for M1-M5 and S1-S2, when diluted by factors of 320, 16, 96, 4, 16, 20, and 48 respectively, fall within the M1 and S1curve ranges. These dilutions were set as low dilution folds to calculate the anti-SARS-CoV-2 WT RBD IgA concentrations of M1-M5 and S1-S2. Dilutions of 5120, 256, 1536, 64, 256, 320, and 768 were set as high dilution folds to calculate the anti-SARS-CoV-2 WT RBD IgA concentrations of M1-M5 and S1-S2, respectively. M1-M5 are NMLF samples (blue), and S1-S2 are serum samples (red).

To evaluate the applicability of serum as a reference for assessing nasal antibodies, ECL with a broad detection range was used to detect SARS-CoV-2 RBD-specific IgA. The highest response values of the nasal samples were similar. In contrast, the highest response values of the nasal samples were twice that of the serum samples (Figure 2(A)). To further understand the impact of this discrepancy on quantitative testing, serum (S1) and nasal (M1) samples were used as the standard to quantify serum and nasal samples. Using S1 as the standard, dilution introduced no bias in the quantitative results of the homologous serum sample; however, it caused a bias for heterologous nasal samples. The IgA concentration of the same nasal sample detected at low dilution was up to 3.8 times that of high dilution (Figure 2(B), Table S6). Similar results were observed when nasal samples were used as standard (Figure 2(C), Table S6). For a heterologous serum sample, the quantitative result of high dilution was up to 10 times that of low dilution (Table S6). This result suggested that using heterologous standards may introduce a bias during the quantification, compromising accuracy. Therefore, the serum-derived standard was unsuitable as a standard for assessing nasal antibodies. Thus, developing a reference standard suitable for assessing nasal antibodies is urgent. Nasal samples or sIgA monoclonal antibody (mAb) may be the suitable material.

### Preparation of CSs using NMLFs or sIgA mAb

To establish CS1 and CS2, we collected NMLFs from 18 healthy donors who recovered from a SARS-CoV-2 infection or received SARS-CoV-2 intranasal vaccine (Table S1). CS1 was a pool of NMLFs collected from 7 donors between May and June 2023, filled as 100 μl/vial. CS2 was a freeze-dried preparation of a pool of NMLFs collected from 11 donors in November 2023. The distributable quantities of CS1 and CS2 were 200 and 1000, respectively. The coefficient of variations (CV) for the anti-XBB.1.5 RBD IgA concentrations of CS1 and CS2 were 12.48%, 4.04%, respectively (Table S2).

Given the challenges in obtaining NMLFs, we also tested if a sIgA mAb could be used as a CS. By mass spectrometry sequencing of nasal spike-specific sIgA combined with single B cell sequencing, we obtained the heavy chain and light chain sequences of a spike-specific mAb (719-1) and expressed its secretory IgA form as 719-1 sIgA. ELISA results showed that 719-1 sIgA exhibited comparable binding activity against various SARS-CoV-2 variants, including WT, Delta, BA.1, BA.5, BA.2.75, BF.7, XBB, and XBB.1.5, with an EC_50_ of 11.5 ± 2.3 ng/ml (Figure S1A). Neutralization data revealed that 719-1 broadly neutralized WT, Delta, XBB.1.5 (Figure S1B). Thus, 719-1 was filled as 2.75 μg/vial and lyophilized. The CV for the anti-XBB.1.5 RBD IgA concentration of CS3 was 2.96%. (Table S2). The residual moistures of CS2 and CS3 were lower than 3%.

### Collaborative study of anti-SARS-CoV-2 nasal IgA standard

This collaborative study was organized by the National Institutes for Food and Drug Control (NIFDC, China). Seven Chinese laboratories experienced in detecting SARS-CoV-2 mucosal IgA participated. These include one national vaccine quality control laboratory, one national laboratory, two vaccine manufacturers, one diagnostic reagent manufacturer, and two research institutes. All laboratories returned their results as required. Laboratories LB1, LB2, LB3, and LB7 returned the in-house or ECL methods results.

#### Validity criteria for parallelism and assay validity

Using ELISA data from laboratories, slope ratios observed for the coded duplicate samples relative to each other (24001 relative to 24004, 24002 relative to 24005, and 24003 relative to 24006) were used to calculate the 95% non-parametric tolerance interval, giving 0.885–1.158. This range was adopted as the acceptable slope ratio range for parallelism.

The data that did not meet linearity and parallelism requirements were excluded to ensure validity. Owing to the decreased binding activity against the JN.1 RBD, CS3 was non-linear and excluded (Table S7). A summary of the exclusions resulting from non-parallelism is shown in Tables S8–S10. Using CS1, CS2, and CS3 as references, the maximum values of non-parallelism for NMLFs were 22.2%, 13.9%, and 12.4%, which were lower than non-parallelism rates for serum sample 24009 (27.8%, 31.2%, and 31.1%). In the ECL method, exclusion severely affected potency calculations 24009 using CSs as reference standards. Due to the different dose–response curves, 24009 failed the non-parallelism check of Combistat software when compared with CSs and was thus excluded (Tables S8–S10). This also indicates significant differences in the properties of the mucosal and serum samples.

#### Binding activity of the CSs

**Figure 3.**
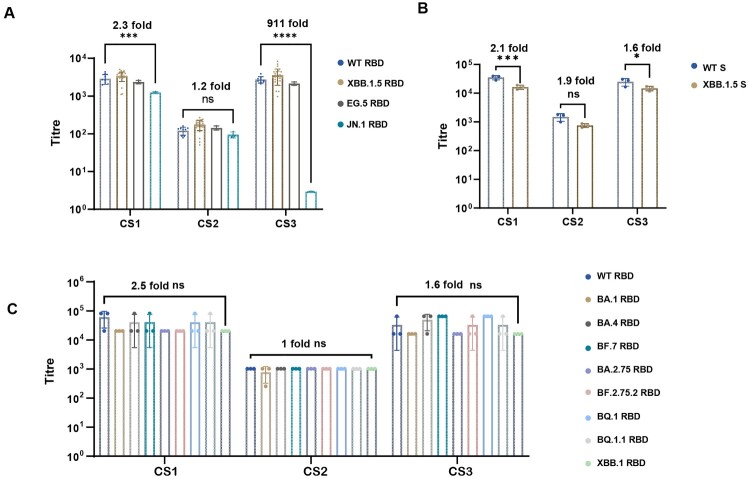
SARS-CoV-2 IgA titres of three candidate standards across all participants. (A) Anti-RBD IgA titres against WT, XBB.1.5, EG.5, and JN.1 using ELISA. (B) Anti-spike IgA titres against WT and XBB.1.5 using ELISA. (C) Anti-RBD IgA titres against WT, BA.1 BA.4, BF.7, BA.2.75, BA.2.75.2, BQ.1 XBB.1 RBD using ECL. Data shown are the means ± SD of the binding antibody titres from three independent experiments, as reported by the participants. Two-way ANOVA is used to analyse the significance of differences in detection results between the latest strain and WT using various detection methods. Significance values are indicated by *P < 0.05, ***P < 0.001; and ****P < 0.0001; ns, not significant.

CS2 exhibited broad-binding activity against the WT and the latest Omicron variant JN.1 (Figure 3). When tested using ELISA, the GMTs of binding antibodies for CS1 and CS2 against the RBD of four strains (WT, XBB.1.5, EG.5, and JN.1) were 1233–3389 and 93–174, respectively, and the spike of two strains (WT and XBB.1.5) were 16231–34838 and 752–1441, respectively. The titres of CS1 were higher than those of CS2. However, the decrease in the binding activity of CS1 against JN.1 RBD compared with that of the WT (2.3 folds) was more significant than that of CS2 (1.2 folds). The ECL detection results also showed that CS2 had broad binding activity against the RBD of WT and early Omicron variants. The binding activity of CS2 to XBB.1 was similar to WT. CS3 showed potent binding activity against WT and early Omicron variants (BA.1, BA.4, BF.7, BA.2.75, BA.2.75.2, BQ.1, BQ.1.1, XBB.1 and XBB.1.5); however its binding activity against JN.1 was significantly decreased . This indicates that using CS3 to evaluate the latest SARS-CoV-2 variants, such as JN.1 is challenging.

#### Intra-assay, intra-laboratory variability in endpoint titres and relative potencies

To evaluate the testing quality of different laboratories, coded duplicate relative potencies for all coded duplicate samples were calculated. Notably, 96% of the relative potency values for duplicate coded samples were within the range of 0.85–1.15, indicating good testing quality in all laboratories (Figure S2).

To demonstrate intra-laboratory variability, the average GCV for collaborative samples within each laboratory was calculated (Table S11). When calculated using the endpoint titres, the GCVs were higher than 20% for some laboratories (Table S11). However, after standardization with the 3 CSs, the GCVs were less than 20%, indicating that the 3 CSs could harmonize intra-laboratory variability.

#### Inter-laboratory variability in endpoint titres and relative potencies

**Figure 4.**
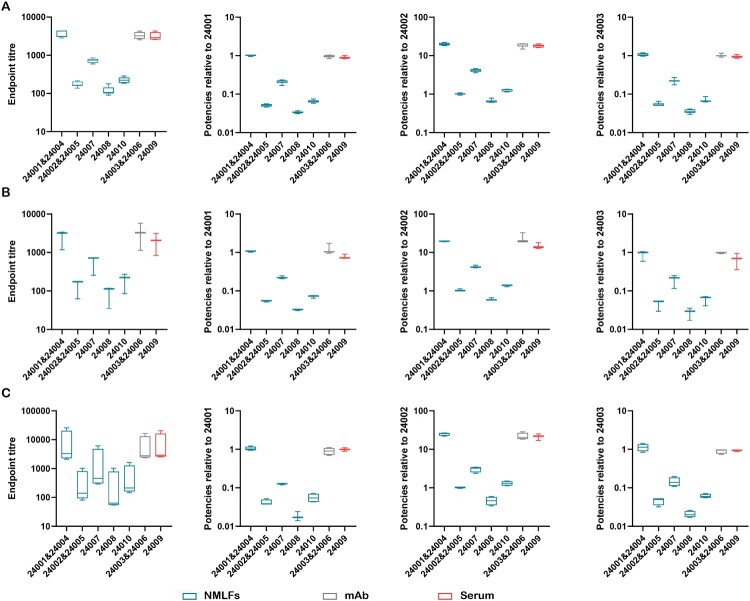
Inter-laboratory variability in endpoint titre and relative potencies. (A) In common methods. (B) XBB.1.5 RBD IgA in-house methods. (C) WT RBD IgA in-house methods. Sample groupings are represented as different colour boxes (blue = NMLFs, grey = mAb, red = serum).

To reflect the impact of the 3 CSs on reducing inter-laboratory variability, the inter-laboratory GCVs of collaborative samples were calculated using 24001 (CS1), 24002 (CS2), and 24003 (CS3) as standards, respectively (Figure 4, Table S12). When calculated using the endpoint titres, the inter-laboratory variability using in-common and in-house methods for XBB.1.5 were 14–26% and 81–127% (Figure 4(A,B), Table S12), and in-house methods for WT showed an inter-laboratory variability of 150–314% (Figure 4(C), Table S12). The GCV of the in-house methods was significantly higher than that of the in-common methods. Standardization with 24001, 24002, and 24003 almost significantly reduced the testing differences among laboratories for all samples. Specifically, standardization with 24002 reduced the GCV for the common method, XBB.1.5 and WT in-house methods to 4–13%, 3–35%, and 4–22%, respectively. Compared with 24002, 24001 remained higher GCVs for the XBB.1.5 and WT in-house methods, at 4–38% and 4–31%, respectively. The mAb-based 24003 had a relatively poor ability to harmonize testing results between different laboratories, with the highest GCVs for the in-common, XBB.1.5 and WT in-house methods of 14%, 64%, and 30%, respectively. In summary, CS2 demonstrated superior capability in harmonizing inter-laboratory variability.

### Assessing the commutability of CS1 and CS2 using regression analysis with a 95% prediction interval (PI)

**Figure 5.**
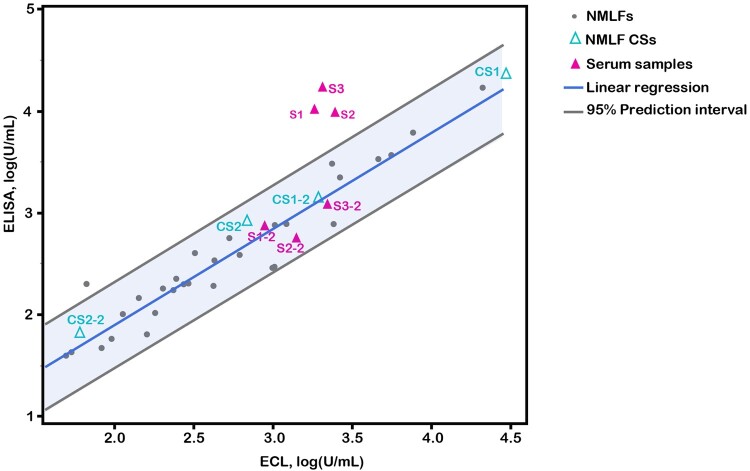
Assessing the commutablity of CS1 and CS2 using regression analysis with a 95% prediction interval. S1, S2, S3 were three serum samples. S1-2, S2-2, S3-2 were S1, S2, S3 with 16-fold diluted. CS1-2 and CS2-2 were CS1 and CS2 with 16-fold diluted.

Commutability, an intrinsic property of standard, is the extent to which the reference standard is suitable for assessing various samples [20, 21]. A non-commutable reference standard could lead to incorrect detection results. Thus, the assessment of commutability is essential. Regression analysis with a 95% PI has been broadly used for assessing commutability [22–24]. The method is based on the results of routine samples and standards with two measurement procedures. A standard is considered commutable if its data point lies within the 95% PI defined by the routine samples. In this study, ECL and ELISA methods were used to assess 30 nasal swabs, CS1, CS2, and serum samples. The results showed that two concentrations of CS1 and CS2 were within the 95% PI of the 30 nasal swabs (Figure 5). In contrast, the higher concentration of serum samples significantly deviated from the 95% PI. These results indicate that CS1 and CS2 were commutable with nasal samples, while serum was non-commutable. Thus, CS1 and CS2 are suitable for detecting nasal samples.

### Assessing the applicability of CS2 with concordance correlation coefficients

To assess consistency between laboratories, concordance correlation coefficients were calculated using log titres or log potencies relative to CS2 for collaborative samples without serum samples (24009). A coefficient value higher than 0.8 indicates good concordance, while a lower value suggests poor concordance [25]. When calculated using endpoint titres, the in-house method of lab 2 targeting XBB.1.5 RBD and the ECL method of lab 7 targeting WT RBD showed poor concordance values (Tables S13 and S14). However, concordance values for all methods improved when expressed relative to CS2 (Tables S15 and S16). This result further demonstrated that the standardization with CS2 can effectively harmonize the differences in detection between different laboratories, ensuring the comparability of test results from different laboratories.

### Evaluation of stability

**Figure 6.**
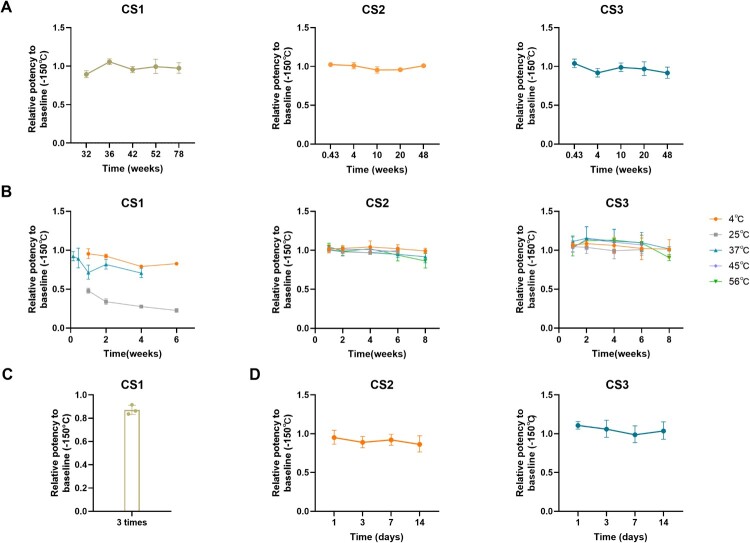
Stability of CSs. (A) Real-time stability of CS1 at −80°C, CS2 and CS3 at −20°C. (B) Accelerated stability of CS1 at three temperatures (4°C, 25°C, and 37°C), CS2 and CS3 at five temperatures (4°C, 25°C, 37°C, 45°C, and 56°C). (C) Freeze-thaw stability of CS1. (D) Reconstituted stability of CS2 and CS3 at 4°C.

The results of real-time stability at the intended storage temperature for all CSs are presented in Figure 6. Relative to the −150°C baseline, the potency of CS1 storage at −80°C for 78 weeks was 0.974. For CS2 and CS3, which were stored at −20°C for 48 weeks, the potencies were 1.009 and 0.919. All CSs were stable at the intended storage temperature.

The CSs’ accelerated stability was conducted by storing samples at elevated temperatures. After 6 weeks at 4 and 25°C and 4 weeks at 37°C, the potencies of CS1 were 0.828, 0.489, and 0.706 folds the baseline samples, respectively (Figure 6(B)). Notably, CS1 had a significant potency decrease at 25°C, even faster than storage at 37°C. The reason needs to be further investigated. The annual degradation rate of CS1 at −80°C or −20°C was 0.34% and 52.41%, respectively. The results suggested that CS1 was stable at −80°C. Freeze-dried formulations CS2 and CS3 showed good stability, with only the samples accelerated at 56°C for 8 weeks, showing a significant decrease (0.864- and 0.904-folds those of the baseline samples). The degradation rate for CS2 stored at −20°C was 1.658% per year. The annual degradation rate of CS3 cannot be calculated, as the data do not conform to the Arrhenius equation.

For liquid CS1, freeze–thaw stability was also investigated. After three freeze–thaw cycles, the antibody in the liquid formulation CS1 was decreased by 13% (Figure 6(C)). Additionally, the reconstitution stability of CS2 and CS3 was conducted. After reconstituting and storing at 4°C for 14 days, the potency of CS3 remained unchanged, while the potency of CS2 decreased by 14%. CS2 should be used immediately after reconstitution (Figure 6(D)). CS2, stable at −20°C, is suitable as a national standard.

## Discussion

The immune response in the respiratory tract is intricate [26]. Mucosal antibodies, cellular immune response, innate immunity, and trained immunity are critical in preventing respiratory pathogen infection [12]. Evaluating the contribution of each type of immunity elicited by respiratory pathogens and mucosal vaccines is essential for identifying strong immunologic correlates of protection against respiratory pathogens, and developing the next-generation mucosal vaccines. Several studies have indicated that SARS-CoV-2 specific nasal IgA but not IgG may serve as an indicator for predicting the risk of re-infection and assessing nasal antibody response after a natural infection or nasal vaccination [14, 16, 27, 28]. Thus, antigen-specific nasal IgA was used as a key indicator for evaluating the effectiveness of mucosal vaccines. Two disparate findings about nasal antibody evaluation were reported recently [29, 30]. The discrepancies may stem from the differences in nasal sample collection methods and immune assays [31]. To ensure the comparability of test results, establishing standardized sampling, immune assays, and standards is urgently required [31, 32].

Standards are benchmarks for unified biological activity values. The availability of biological material for antibodies would accelerate the standardization of immune assays and facilitate the comparison and harmonization of datasets among different laboratories. Serum standards have mainly been used to assess mucosal antibodies [14, 27]. However, considering the differences in Ig composition, function, and activity, serum may be an unsuitable standard for mucosal antibodies [15, 16, 33]. Based on systematic studies, we extensively validated this hypothesis. First, the different Ig compositions in serum and nasal samples were confirmed (Figure 1). Subsequently, significant differences in the dose–response curves were observed, using ECL to assess serum and nasal samples (Figure 2(A)). The upper platform response value of the nasal samples was twice that of the serum samples. Applying heterologous standards for quantification, the results of various dilutions of the identical sample exhibited significant variations (Figure 2(B,C)). The variations can be 10 times (Table S6). Meanwhile, the different curves also led to a higher rate of non-parallel exclusion of serum samples in collaborative studies (Tables S8–S10). Additionally, we observed that the ability of nasal standards to reduce the inter-laboratory GCVs for serum samples was substantially weaker than that of nasal samples (Figure 4). A similar result was observed in a collaborative study of the national standard for XBB.1.5 neutralizing antibodies [34]. To further confirm the differences in commutability between nasal and serum samples, the commutability assessment guidelines were referenced for related studies [22, 35]. The results showed that high concentrations of serum samples significantly deviated from the 95% PI of nasal samples (Figure 5). Thus, serum standards have poor applicability for detecting NMLFs. Through systematic studies, significant differences in binding characteristics between NMLFs and serum samples were discovered for the first time. Our studies demonstrated the rationality of the WHO standard material development guidelines for standard raw materials requirements [21]. Moreover, we highlight the significance of commutability in developing antibody reference standards. However, owing to the different types and characteristics of biological reference materials, the methods for commutability studies may also differ [24]. Therefore, targeted commutability studies for different types of biological reference materials require further strengthening.

In this study, NMLFs from COVID-19 convalescents and mucosal vaccine recipients were first used as raw materials. The respiratory mucosa directly interacts with the external environment and is colonized by various microorganisms, including *Staphylococcus*, *Propionibacterium*, and *Streptococcus* [36]. Some microorganisms can cause hypo-immunity-related diseases. To ensure standard safety, NMLFs were collected from healthy donors and assessed for 14 respiratory pathogens. After screening for safety and IgA activity, CS1 and CS2 were prepared. In addition, a highly potent sIgA mAb binding to the spike of SARS-CoV-2 was used to prepare CS3 for subsequent collaborative study.

7 laboratories participated in the collaborative study. In the initial phases of our study, XBB sublineages were the predominant strains. Thus, a common ELISA method targeting XBB.1.5 RBD was provided for all laboratories. 7 in-house ELISA methods targeting WT, XBB.1.5, EG.5, and JN.1 RBD, and 2 in-house ELISA methods targeting WT and XBB.1.5 spike were also included. Additionally, a commercial ECL-RBD method, covering 9 strains from WT to XBB.1, was used. Although CS3 showed good broad-spectrum binding activity against RBD from WT to EG.5, its binding activity against JN.1 significantly decreased (Figure 3). Standardized with CS3, the inter-laboratory variabilities for most samples were reduced. However, the harmonization achieved with CS3 was poorer than using CS1 and CS2, especially in the method targeting WT RBD (Table S12). Similar results were obtained in the first national standard for XBB neutralization antibody [34]. WHO has prepared two mAb mixtures using 7 and 4 anti-Lassa virus mAbs, respectively [37]. Collaborative studies revealed that some laboratories tested negative for these mixtures, and their ability to harmonize inter-laboratory differences remained weaker than that of serum mixtures. A mAb cocktail containing over 7 mAbs may be suitable as a reference material. However, this hypothesis needs further validation.

To ensure the stability of the standard materials, this study prepared the liquid (CS1) and freeze-dried preparations (CS2, CS3). Stability studies showed that the freeze-dried CS2 and CS3 had good real-time and accelerated stability. The Arrhenius equation revealed the annual degradation rate of CS2 stored at −20°C to be 1.658%. The potency of CS2 decreased rapidly after reconstitution, indicating that it should be used immediately after reconstitution. Notably, the degradation rate of XBB.1.5 RBD IgA by CS1 at 25°C was faster than that at 37°C. Given the diversity of enzymes and foreign microorganisms in the respiratory mucosa, it was speculated that some proteases in the raw materials of CS1 may degrade IgA [38]. however, this requires further investigation.

This study pioneered the use of NMLFs as the raw material to establish the 1st national standard for anti-SARS-CoV-2 nasal IgA (Lot: 300052-202401, 1000 U/ml), which has demonstrated good safety, stability, consistency, and broad binding activity against WT-JN.1. However, data presented in our study cannot be used to define the regulatory requirements for mucosal vaccines. Using nasal standards effectively harmonizes anti-SARS-CoV-2 nasal antibody results across different laboratories and methods and facilitates more studies to collect data on nasal IgA. Thus, the understanding of mucosal immunity would be advanced, and the mucosal correlates of protection could be determined.

## Supplementary Material

Supplementary no revision.docx
