# Supplementary material for: Exploring the standardization of human nasal antibody measurements
Source: Emerg Microbes Infect. 2025 Mar 12;14(1):2475822. doi: 10.1080/22221751.2025.2475822 (PMC11915745; doi:10.1080/22221751.2025.2475822)

**Supplementary**

**Table S1 Clinical information of NMLF donors**

| **Code** | **COVID-19 vaccination status** | **Months from the last priming dose** | **Months from prior infection** |  |
| --- | --- | --- | --- | --- |
| **NMLFs used for pooling CS1** | | | | |
| 1 | 3 doses of inactivated vaccine, 3 doses of intranasal adenovirus-vectored vaccine | 3 | Uninfected |  |
| 2 | 1 dose of intranasal adenovirus-vectored vaccine | 3 | 3 |  |
| 3 | 2 doses of inactivated vaccine, 1 dose of intranasal adenovirus-vectored vaccine | 10 | Uninfected |  |
| 4 | 3 doses of inactivated vaccine, 3 doses of intranasal adenovirus-vectored vaccine | 1 | 6 |  |
| 5 | 3 doses of inactivated vaccine, 2 doses of intranasal adenovirus-vectored vaccine | 6 | 1 |  |
| 6 | 2 doses of inactivated vaccine, 4 doses of intranasal adenovirus-vectored vaccine | 10 | Uninfected |  |
| 7 | 4 doses of intranasal adenovirus-vectored vaccine | 1 | Uninfected |  |
| **NMLFs used for pooling CS2** | | | | |
| 1 | 3 doses of inactivated vaccine | 22 | Uninfected |  |
| 2 | 3 doses of recombinant vaccine | 24 | 6 |  |
| 3 | 3 doses of inactivated vaccine | 19 | 6 |  |
| 4 | 3 doses of inactivated vaccine, 1 dose of intranasal adenovirus-vectored vaccine | 11 | 11 |  |
| 5 | 2 doses of inactivated vaccine, 2 doses of intranasal adenovirus-vectored vaccine | 10 | 10 |  |
| 6 | 3 doses of inactivated vaccine | 23 | 6 |  |
| 7 | 3 doses of inactivated vaccine, 3 doses of intranasal adenovirus-vectored vaccine | 9 | Uninfected |  |
| 8 | 4 doses of intranasal adenovirus-vectored vaccine | 6 | 2 |  |
| 9 | 3 doses of inactivated vaccine, 3 doses of intranasal adenovirus-vectored vaccine | 9 | Uninfected |  |
| 10 | 2 doses of inactivated vaccine, 7 doses of intranasal adenovirus-vectored vaccine | 6 | 1 |  |
| 11 | 3 doses of inactivated vaccine, 2 doses of intranasal adenovirus-vectored vaccine | 11 | 6 |  |

**Table S2. Summary of CSs**

| Code | CS1 | CS2 | CS3 |
| --- | --- | --- | --- |
| Formulation | Liquid | Freeze-dry | Freeze-dry |
| CV of anti-XBB.1.5 RBD IgA | 12.48% | 4.04% | 2.96% |
| CV of fill mass | 0.2% | 0.4% | 0.2% |
| Residual moisture | / | 2.829% | 1.280% |
| Storage temperature | -80℃ | -20℃ | -20℃ |
| Vials available for distribution | 200 | 1000 | 700 |

**Table S3. Samples included in the collaborative study**

| Sample code | Description | Formulation |
| --- | --- | --- |
| 24001, 24004 | Candidate 1, nasal washes collected from 7 donors in May 2023. | Liquid |
| 24002, 24005 | Candidate 2, nasal washes collected from 11 donors in November 2023. | Freeze-dry |
| 24003, 24006 | Candidate 3, sIgA-719-1, a monoclonal antibody cloned from the nasal mucosa. | Freeze-dry |
| 24007 | Nasal wash from one donor with a high titer of XBB.1.5 RBD IgA, collected in January 2024 | Liquid |
| 24008 | Nasal wash from one donor with a low titer of XBB.1.5 RBD IgA, collected in January 2024 | Liquid |
| 24009 | Candidate standard deviated from XBB convalescent plasma. | Liquid |
| 24010 | Nasal wash was collected from donors between November and December 2023, with the addition of sIgA-719-1. | Freeze-dry |
| 20/136 | The First WHO International Standard for anti-SARS-CoV-2  Immunoglobulin, derived from plasma in 2020. For binding antibody assays, an arbitrary unitage of 1000 binding antibody units (BAU)/mL can be used to assist the comparison of assays detecting the same class of immunoglobulins with the same specificity. This standard was only used in Lab 7 for MSD. | Freeze-dry |

**Table S4 List of collaborative study participants**

| NIFDC (Beijing, China) |
| --- |
| Guangzhou Laboratory (Guangzhou, China) |
| Guangzhou Institutes of Biomedicine and Health (Guangzhou, China) |
| Beijing Wantai Biological Pharmacy Enterprise, Ltd. (Beijing, China) |
| CanSino Biologics Inc. (Tianjin, China) |
| Vazyme Biotech Co., Ltd. (Nanjing, China) |
| Sun Yat-sen University (Shenzhen, China) |

**Table S5. Assays used in the collaborative study**

| Type of assay | In-common/ in-house/commercial | Antigen | Readout | Participant code |
| --- | --- | --- | --- | --- |
| Enzyme immunoassays  （ELISA） | In-common | XBB.1.5 RBD | O.D. | 1,2,3,4,5,6,7 |
|  | In-house | XBB.1.5 RBD | O.D. | 2,3 |
|  | In-house | WT RBD | O.D. | 1,2,3 |
|  | In-house, | EG.5 RBD | O.D. | 7 |
|  | In-house, | JN.1 RBD | O.D. | 7 |
|  | In-house, | WT S | O.D. | 3 |
|  | In-house, | XBB.1.5 S | O.D. | 3 |
| Electrochemical luminescence  （ECL） | V-PLEX SARS-CoV-2 Panel 33 Kit (Meso Scale Discovery) | WT, BA.1, BQ.1.1, BF.7, BA.2.75.2, BA.2.75, BQ.1, XBB.1 BA.4/5 RBD | RLU | 7 |

O.D.: optical density; RLU: relative luminescence units

**Table S6. The anti-SARS-CoV-2 WT RBD IgA concentration of serum and mucosal samples in low and high dilution.**

| **Sample code** | **S1(Serum standard)** | | | | **M1 (Mucosal standard)** | | |
| --- | --- | --- | --- | --- | --- | --- | --- |
|  | **Low dilution（U/ml）** | **High dilution**  **（U/ml）** | **Low/High** | **Low dilution（U/ml）** | | **High dilution**  **（U/ml）** | **Low/High** |
| M1 | 49 | 29 | 1.69 | / | | / | / |
| M2 | 3531 | 930 | 3.80 | 29473 | | 30868 | 0.95 |
| M3 | 131 | 114 | 1.15 | 3471 | | 3790 | 0.92 |
| M4 | 29 | 12 | 2.39 | 332 | | 397 | 0.84 |
| M5 | 85 | 39 | 2.21 | 1208 | | 1283 | 0.94 |
| S1 | / | / | / | 2045 | | 19896 | 0.10 |
| S2 | 194 | 162 | 1.20 | 5970 | | 18669 | 0.32 |

The anti-SARS-CoV-2 WT RBD IgA concentrations of M1 and S1 were defined as 1000 U/ml. Detection values for M1-M5 and S1-S2, when diluted by factors of 320, 16, 96, 4, 16, 20, and 48 respectively, fall within the M1 and S1curve ranges. These dilutions are used to calculate low concentrations. For high concentrations, dilutions of 5120, 256, 1536, 64, 256, 320, and 768 are used respectively.

**Table S7. Exclusions due to non-linearity for study samples**

| **Method** | **Targeted antigen** | **Sample code** | | | | | | | | | | |
| --- | --- | --- | --- | --- | --- | --- | --- | --- | --- | --- | --- | --- |
|  |  | **24001** | **24002** | **24003** | **24004** | **24005** | **24006** | **24007** | **24008** | **24009** | **24010** |  |
| In common ELISA | XBB.1.5 RBD | 0 | 0 | 0 | 0 | 0 | 0 | 0 | 0 | 0 | 0 |  |
| In house ELISA | XBB.1.5 RBD | 0 | 0 | 0 | 0 | 0 | 0 | 0 | 0 | 0 | 0 |  |
|  | WT RBD | 0 | 0 | 0 | 0 | 0 | 0 | 0 | 0 | 0 | 0 |  |
|  | EG.5 RBD | 0 | 0 | 0 | 0 | 0 | 0 | 0 | 0 | 0 | 0 |  |
|  | JN.1 RBD | 0 | 0 | 100% | 0 | 0 | 100% | 0 | 0 | 0 | 0 |  |
|  | XBB.1.5 S | 0 | 0 | 0 | 0 | 0 | 0 | 0 | 0 | 0 | 0 |  |
|  | WT S | 0 | 0 | 0 | 0 | 0 | 0 | 0 | 0 | 0 | 0 |  |
| Total |  | 0 | 0 | 14.3% | 0 | 0 | 14.3% | 0 | 0 | 0 | 0 |  |

**Table S8. Exclusions due to non-parallelism for study samples, using 24001 as a reference**

|  |  | **Sample code** | | | | | | | | | | |
| --- | --- | --- | --- | --- | --- | --- | --- | --- | --- | --- | --- | --- |
| **Method** | **Targeted antigen** | 24001 | 24002 | 24003 | 24004 | 24005 | 24006 | 24007 | 24008 | 24009 | 24010 |  |
| In common ELISA | XBB.1.5 RBD | / | 4.8% | 0 | 0 | 4.8% | 0 | 4.8% | 0 | 0 | 0 |  |
| In house ELISA | XBB.1.5 RBD | / | 0 | 0 | 0 | 0 | 16.7% | 16.7% | 16.7% | 0 | 33.3% |  |
|  | WT RBD | / | 0 | 0 | 0 | 0 | 11.1% | 44.4% | 66.7% | 55.6% | 44.4% |  |
|  | EG.5 RBD | / | 0 | 0 | 0 | 0 | 0 | 0 | 0 | 0 | 0 |  |
|  | JN.1 RBD | / | 0 | / | 0 | 0 | / | 33.3% | 0 | 0 | 33.3% |  |
|  | XBB.1.5 S | / | 0 | 0 | 0 | 0 | 0 | 0 | 0 | 0 | 33.3% |  |
|  | WT S | / | 0 | 0 | 0 | 0 | 0 | 0 | 33.3% | 66.7% | 33.3% |  |
| MSD | WT RBD | / | 0 | 0 | 0 | 0 | 0 | 0 | 0 | 100% | 0 |  |
| Total |  |  | 0.6% | 0 | 0 | 0.6% | 3.5% | 12.4% | 14.5% | **27.8%** | 22.2% |  |

**Table S9. Exclusions due to non-parallelism for study samples, using 24002 as a reference**

|  |  | **Sample code** | | | | | | | | | | |
| --- | --- | --- | --- | --- | --- | --- | --- | --- | --- | --- | --- | --- |
| **Method** | **Targeted antigen** | 24001 | 24002 | 24003 | 24004 | 24005 | 24006 | 24007 | 24008 | 24009 | 24010 |  |
| In common ELISA | XBB.1.5 RBD | 4.8% | / | 4.8% | 0 | 4.8% | 4.8% | 4.8% | 0 | 4.8% | 0 |  |
| In house ELISA | XBB.1.5 RBD | 0 | / | 0 | 0 | 0 | 16.7% | 16.7% | 16.7% | 0 | 33.3% |  |
|  | WT RBD | 0 | / | 0 | 0 | 0 | 11.1% | 11.1% | 33.3% | 44.4% | 44.4% |  |
|  | EG.5 RBD | 0 | / | 0 | 0 | 0 | 0 | 0 | 0 | 33.3% | 0 |  |
|  | JN.1 RBD | 0 | / | / | 0 | 0 | / | 33.3% | 0 | 0 | 33.3% |  |
|  | XBB.1.5 S | 0 | / | 0 | 0 | 0 | 0 | 0 | 0 | 0 | 0 |  |
|  | WT S | 0 | / | 0 | 0 | 0 | 0 | 0 | 0 | 66.7% | 0 |  |
| MSD | WT RBD | 0 | / | 0 | 0 | 0 | 0 | 0 | 0 | 100% | 0 |  |
| Total |  | 0.6% | / | 0.6% | 0 | 0.6% | 4.1% | 8.2% | 6.3% | **31.2%** | 13.9% |  |

**Table S10. Exclusions due to non-parallelism for study samples, using 24003 as a reference**

|  |  | **Sample code** | | | | | | | | | | |
| --- | --- | --- | --- | --- | --- | --- | --- | --- | --- | --- | --- | --- |
| **Method** | **Targeted antigen** | 24001 | 24002 | 24003 | 24004 | 24005 | 24006 | 24007 | 24008 | 24009 | 24010 |  |
| In common ELISA | XBB.1.5 RBD | 4.8% | 0 | / | 0 | 4.8% | 0 | 4.8% | 4.8% | 4.8% | 4.8% |  |
| In house ELISA | XBB.1.5 RBD | 16.7% | 16.7% | / | 0 | 16.7% | 0 | 16.7% | 0 | 33.3% | 16.7% |  |
|  | WT RBD | 11.1% | 11.1% | / | 11.1% | 11.1% | 22.2% | 11.1% | 33.3% | 44.4% | 44.4% |  |
|  | EG.5 RBD | / | / | / | 0 | 0 | 0 | 0 | 0 | 33.3% | 33.3% |  |
|  | JN.1 RBD | / | / | / | / | / | / | / | / | / | / |  |
|  | XBB.1.5 S | 0 | 0 | / | 0 | 0 | 0 | 0 | 0 | 0 | 0 |  |
|  | WT S | 0 | 0 | / | 0 | 0 | 0 | 0 | 0 | 33.3% | 0 |  |
| MSD | WT RBD | 0 | 0 | / | 0 | 0 | 0 | 0 | 0 | 100% | 0 |  |
| Total |  | 4.1% | 3.5% | / | 1.4% | 4.1% | 2.8% | 4.1% | 4.8% | 31.1% | 12.4% |  |

**Table S11. Summary of intra-laboratory (between assay) variability for study samples: Average GCV**

|  | In common  XBB.1.5 RBD | | | | | | | In house  XBB.1.5 RBD | | In house  WT RBD | | | |
| --- | --- | --- | --- | --- | --- | --- | --- | --- | --- | --- | --- | --- | --- |
| Lab code | 1 | 2 | 3 | 4 | 5 | 6 | 7 | 2 | 3 | 1 | 2 | 3 | 7 |
| Titer | 10% | 21% | 23% | 13% | 15% | 5% | 9% | 29% | 28% | 5% | 8% | 23% | 15% |
| RT/24001 | 3% | 4% | 8% | 8% | 14% | 3% | 5% | 8% | 3% | 1% | 9% | 11% | 5% |
| RT/24002 | 4% | 4% | 13% | 11% | 5% | 5% | 5% | 7% | 5% | 3% | 10% | 8% | 3% |
| RT/24003 | 6% | 6% | 12% | 11% | 4% | 6% | 5% | 18% | 9% | 3% | 13% | 6% | 5% |

Black represents the GCV of end-point titers. Blue represents a lower GCV of relative titers than that of endpoint titers. Red represents equal or higher GCV of relative titers than that of endpoint titers.

**Table S12 Summary of inter-laboratory GCV value**

| Name of Assay and target antigen | Sample code | 24001&  24004 | 24002&  24005 | 24003&  24006 | 24007 | 24008 | 24009 | 24010 |
| --- | --- | --- | --- | --- | --- | --- | --- | --- |
| In common, XBB.1.5 RBD | Titer | 21% | 18% | 20% | 14% | 26% | 23% | 18% |
|  | RT/24001 | 4% | 8% | 8% | 12% | 7% | 7% | 9% |
|  | RT/24002 | 8% | 4% | 13% | 9% | 9% | 8% | 7% |
|  | RT/24003 | 8% | 10% | 7% | 14% | 11% | 8% | 12% |
| In house,  XBB.1.5 RBD | Titer | 81% | 81% | 127% | 82% | 100% | 96% | 85% |
|  | RT/24001 | 4% | 5% | 38% | 9% | 4% | 14% | 9% |
|  | RT/24002 | 3% | 7% | 35% | 8% | 8% | 20% | 5% |
|  | RT/24003 | 38% | 40% | 4% | 51% | 46% | 64% | 36% |
| In house,  WT RBD | Titer | 209% | 206% | 150% | 308% | 314% | 172% | 198% |
|  | RT/24001 | 11% | 17% | 23% | 4% | 31% | 16% | 29% |
|  | RT/24002 | 9% | 4% | 22% | 18% | 28% | 22% | 14% |
|  | RT/24003 | 24% | 28% | 15% | 30% | 20% | 6% | 12% |

Black represents the GCV of end-point titers. Blue represents a lower GCV of relative titers than that of endpoint titers. Red represents equal or higher GCV of relative titers than that of endpoint titers.

**Table S13. Concordance correlation coefficients for anti-XBB.1.5 RBD IgA log titer excluding serum sample 24009.**

|  |  | In common | | | | | | | In house | |
| --- | --- | --- | --- | --- | --- | --- | --- | --- | --- | --- |
|  | Lab | 1 | 2 | 3 | 4 | 5 | 6 | 7 | 2 | 3 |
| In common | 1 | - |  |  |  |  |  |  |  |  |
|  | 2 | 0.94 |  |  |  |  |  |  |  |  |
|  | 3 | 0.98 | 0.99 |  |  |  |  |  |  |  |
|  | 4 | 1.00 | 0.96 | 0.99 |  |  |  |  |  |  |
|  | 5 | 0.96 | 1.00 | 1.00 | 0.98 |  |  |  |  |  |
|  | 6 | 0.97 | 0.99 | 1.00 | 0.99 | 1.00 |  |  |  |  |
|  | 7 | 0.96 | 0.99 | 1.00 | 0.98 | 1.00 | 1.00 |  |  |  |
| In house | 2 | 0.68 | 0.83 | 0.77 | 0.71 | 0.81 | 0.79 | 0.79 |  |  |
|  | 3 | 0.98 | 0.97 | 0.98 | 0.99 | 0.98 | 0.99 | 0.97 | 0.74 |  |

Values 0.8~0.9 were shaded with green, >0.9 were shaded with red.

**Table S14. Concordance correlation coefficients for anti-WT RBD IgA log titer excluding serum sample 24009.**

| Lab | 1 | 2 | 3 | 7 |
| --- | --- | --- | --- | --- |
| 1 |  |  |  |  |
| 2 | 0.98 |  |  |  |
| 3 | 0.99 | 0.95 |  |  |
| 7 | 0.40 | 0.36 | 0.45 |  |

Values 0.8~0.9 were shaded with green, >0.9 were shaded with red.

**Table S15. Concordance correlation coefficients for anti-XBB.1.5 RBD IgA log potency relative to 24002 excluding serum sample 24009.**

|  |  | In common | | | | | | | In house | |
| --- | --- | --- | --- | --- | --- | --- | --- | --- | --- | --- |
|  | Lab | 1 | 2 | 3 | 4 | 5 | 6 | 7 | 2 | 3 |
| In common | 1 |  |  |  |  |  |  |  |  |  |
|  | 2 | 1.00 |  |  |  |  |  |  |  |  |
|  | 3 | 1.00 | 1.00 |  |  |  |  |  |  |  |
|  | 4 | 1.00 | 1.00 | 1.00 |  |  |  |  |  |  |
|  | 5 | 0.99 | 1.00 | 1.00 | 1.00 |  |  |  |  |  |
|  | 6 | 1.00 | 1.00 | 1.00 | 1.00 | 1.00 |  |  |  |  |
|  | 7 | 0.99 | 0.99 | 0.99 | 1.00 | 1.00 | 1.00 |  |  |  |
| In house | 2 | 0.99 | 1.00 | 0.99 | 0.99 | 1.00 | 1.00 | 0.99 |  |  |
|  | 3 | 0.98 | 0.99 | 0.98 | 0.99 | 0.98 | 0.99 | 0.97 | 0.99 |  |

Values 0.8~0.9 were shaded with green, >0.9 were shaded with red.

**Table S16. Concordance correlation coefficients for anti-WT RBD IgA log potency relative to 24002 excluding serum sample 24009.**

| Lab | 1 | 2 | 3 | 7 |
| --- | --- | --- | --- | --- |
| 1 |  |  |  |  |
| 2 | 0.98 |  |  |  |
| 3 | 0.99 | 0.99 |  |  |
| 7 | 0.99 | 0.99 | 1.00 |  |

Values 0.8~0.9 were shaded with green, >0.9 were shaded with red.

Figure S1. Analysis of the binding and neutralizing activities of 719-1 A: The binding activities of 719-1 against spikes of Omicron subvariants (BA.1, BA.5, BA.2.74, BF.7, XBB, XBB.1.5), pre-Omicron WT and Delta. The results are presented as 50% effective concentration (EC_50_) in nM. B: Pseudovirus NtAb of 719-1 against WT, Delta and XBB.1.5. The results are presented as 50% inhibitory concentration (IC_50_) in nM.

Figure S2. Intra-assay variability. A: Relative potencies of coded duplicate samples 24001 and 24004 with in common method and XBB.1.5 RBD IgA in house method. B: Relative potencies of coded duplicate samples 24002 and 24005 with in common method and XBB.1.5 RBD IgA in house method. C: Relative potencies of coded duplicate samples 24003 and 24006 with in common method and XBB.1.5 RBD IgA in house method.

Figure S1


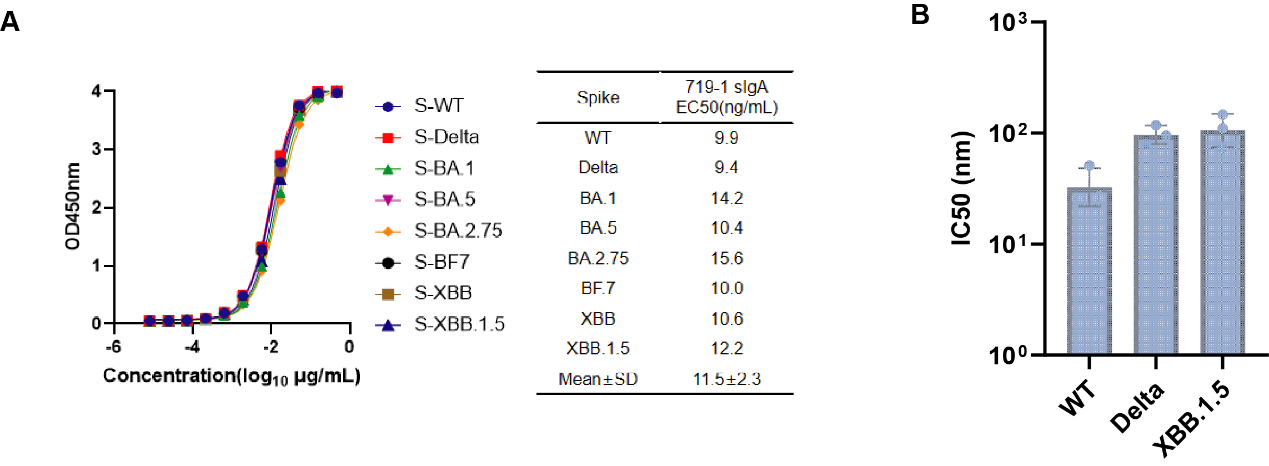


Figure S2


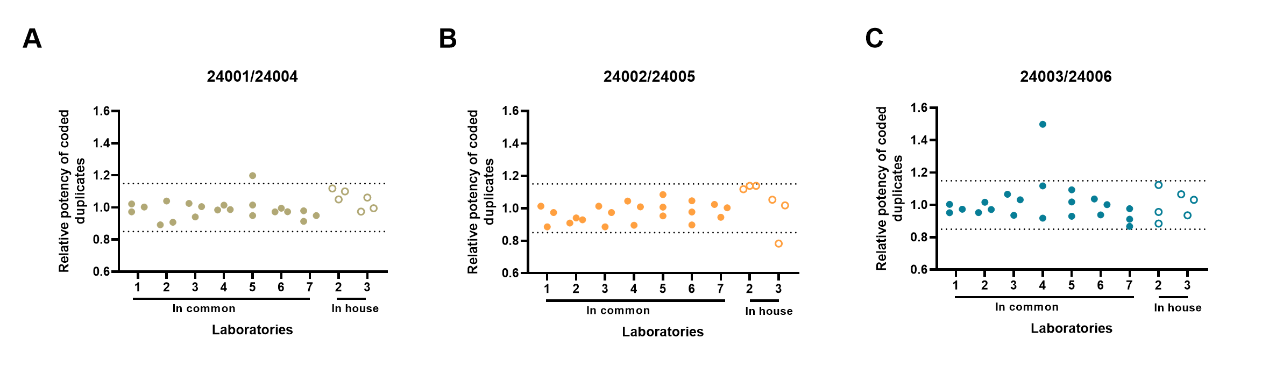

Supplement: Supplementary no revision.docx [file TEMI_A_2475822_SM8548.docx]
